# P-2157. Comparative Analysis of Sepsis Outcomes in Patients with Left Ventricular Assist Devices versus Heart Transplantation: A National Database Analysis

**DOI:** 10.1093/ofid/ofaf695.2320

**Published:** 2026-01-11

**Authors:** Sarim Raheel, Zulfiqar H Jogezai, Muskaan Abdul Qadir, Hafsa Khan Tareen, Bilal Siddiqui, Ayesha Rashid, Armaghan-e-Rehman Mansoor

**Affiliations:** Aga Khan University, Karachi, Sindh, Pakistan; The Aga Khan University, New York City, New York; University of New Mexico, Albuquerque, NewMexico; Aga Khan University, Karachi, Sindh, Pakistan; Midwestern University, Naperville, Illinois; Allina Health, University of Minnesota, St. Paul, Minnesota; University of Kentucky, Lexington, KY

## Abstract

**Background:**

Heart transplantation and Left Ventricular Assist Devices (LVAD) are treatment options for end-stage heart failure. Infectious complications are common among both groups, however there is a paucity of data evaluating the burden of sepsis in these cohorts. This study aims to compare the incidence, epidemiology and outcomes of inpatient admissions for sepsis in patients with LVADs versus heart transplant.

**Table 1:**
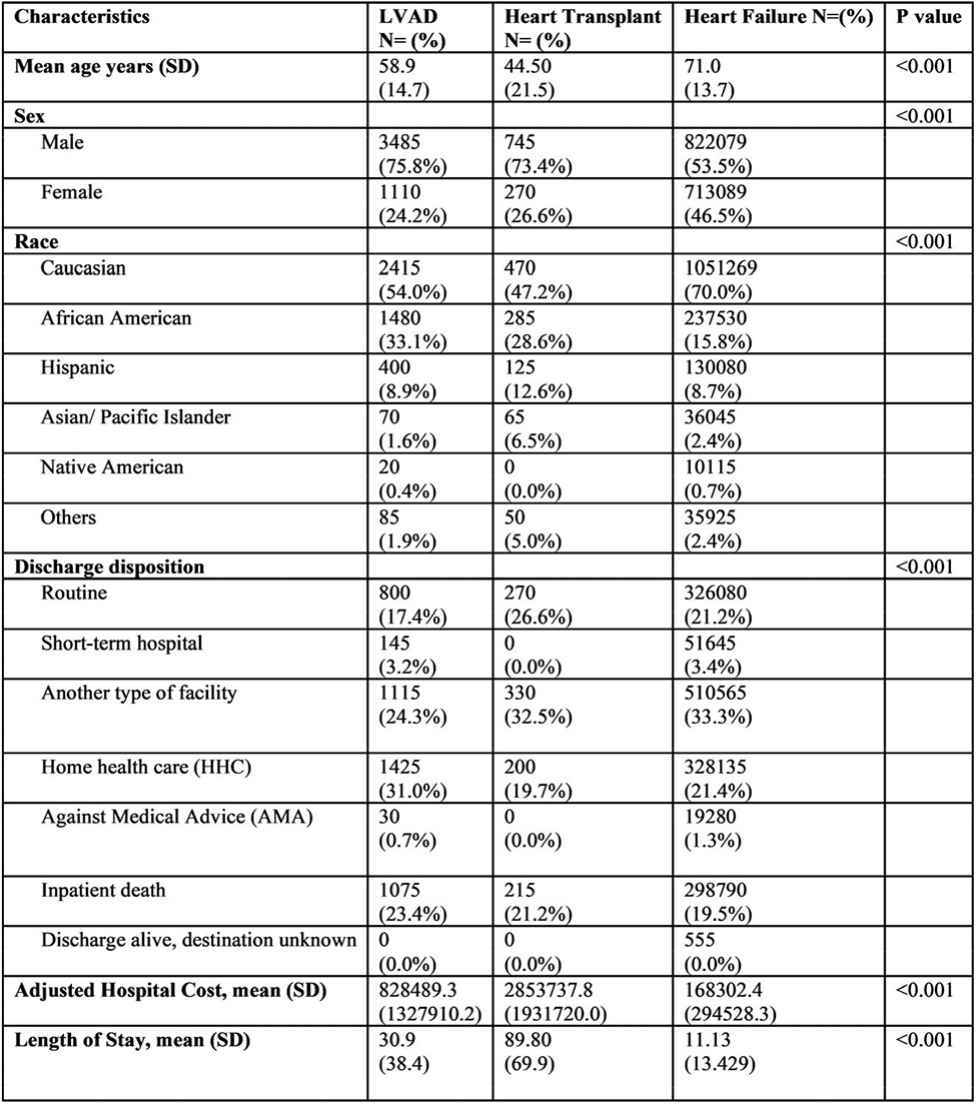
Demographics and Characteristics of patients with Sepsis; LVAD: left ventricular assist device

**Table 2:**
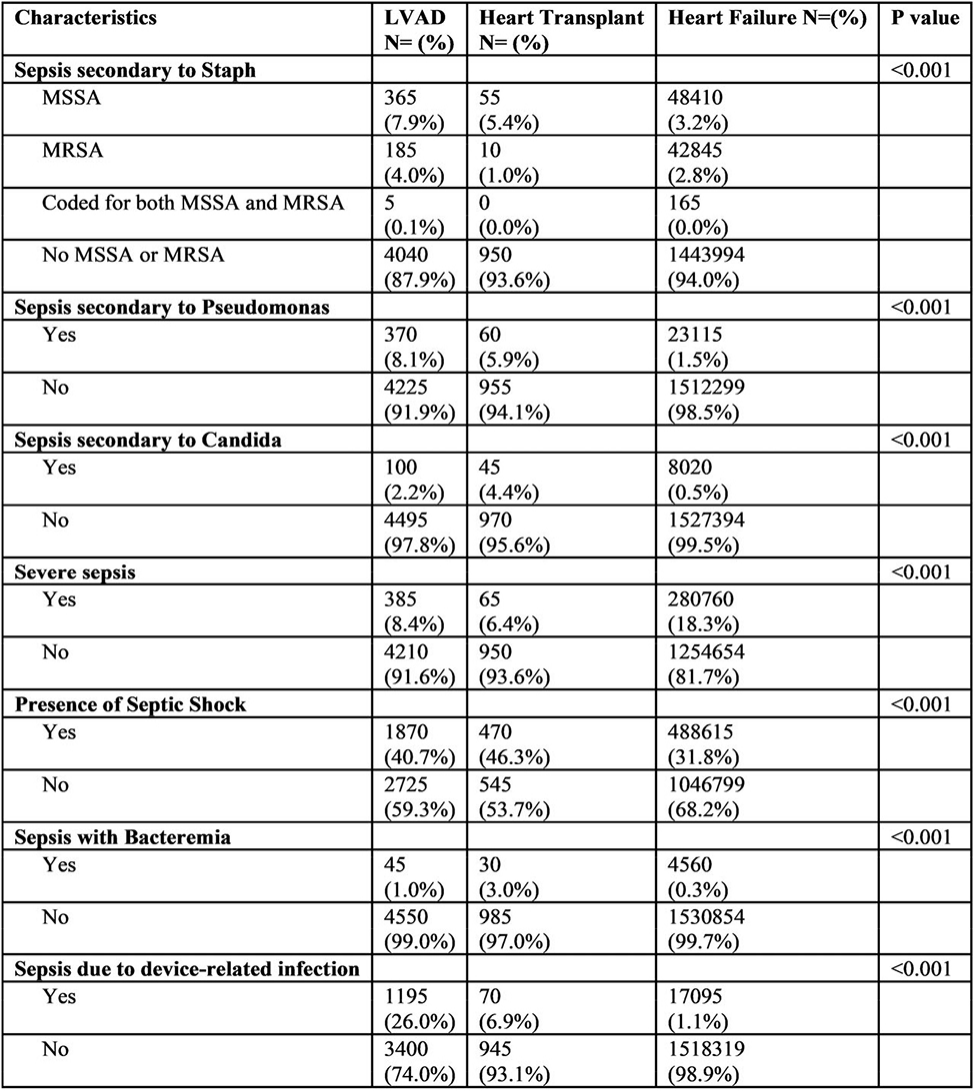
Etiology of Sepsis in comparator groups; LVAD: left ventricular assist device

**Methods:**

The National Inpatient Sample (NIS) was queried for inpatient admissions with a primary diagnosis of sepsis or severe sepsis between January 2021 to December 2022. Patients with an LVAD or history of heart transplantation were identified using ICD-10 procedure and diagnosis codes. Patients with heart failure without a history of LVAD implantation or heart transplant served as the control group. Demographics, outcomes, in-hospital mortality, length of stay, disposition, and total hospitalization costs were analyzed using SPSS (v. 30, IBM). Weights assigned in NIS were used to calculate national estimates.

**Results:**

A total of 1,541,024 sepsis admissions were analyzed, including 4,595 LVAD and 1,015 heart transplant recipients (Table 1). Heart transplant recipients were younger (mean 44.5 vs. 58.9 [LVAD] vs. 71.0 years [HF], p< 0.001). LVAD patients had the highest in-hospital mortality (23.4% vs. 21.2% [transplant] vs. 19.5% [HF], p< 0.001). Transplant patients had longer hospital stays (mean 89.8 vs. 30.9 vs. 13.7 days) and higher adjusted costs ($2.85M vs. $828K vs. $197K; p< 0.001). Severe sepsis was more frequent in HF patients (18.3%), while septic shock (46.3%) and bacteremia (3.0%) were more common in transplant recipients (p< 0.001) (Table 2). LVAD patients had higher rates of device-related infections (26.0%) and Pseudomonal sepsis (8.1%), whereas transplant recipients had higher rate of Candidal sepsis (4.4%) (p< 0.001).

**Conclusion:**

LVAD and heart transplant recipients experienced significantly higher mortality, longer hospital stays, and higher inpatient costs compared to medically managed heart failure patients. Given their unique infectious risks—device-related in LVADs and immunosuppression-related in transplant recipients—clinicians must adopt vigilant, cohort-specific strategies to mitigate the infection risks in these populations.

**Disclosures:**

All Authors: No reported disclosures

